# Anti-inflammatory Effect of Curcumin, Homotaurine, and Vitamin D3 on Human Vitreous in Patients With Diabetic Retinopathy

**DOI:** 10.3389/fneur.2020.592274

**Published:** 2021-02-05

**Authors:** Mariaelena Filippelli, Giuseppe Campagna, Pasquale Vito, Tiziana Zotti, Luca Ventre, Michele Rinaldi, Silvia Bartollino, Roberto dell'Omo, Ciro Costagliola

**Affiliations:** ^1^Department of Medicine and Health Sciences “V. Tiberio”, University of Molise, Campobasso, Italy; ^2^Department of Medical-Surgical Sciences and Translational Medicine, University of Rome “La Sapienza”, Rome, Italy; ^3^Sannio Tech Consortium, Apollosa, Italy; ^4^Department of Science and Technology, University of Sannio, Benevento, Italy; ^5^Azienda Ospedaliero-Universitaria Città della Salute e della Scienza di Torino, University Eye Clinic, Turin, Italy; ^6^Multidisciplinary Department of Medical, Surgical and Dental Sciences, Eye Clinic, University of Campania Luigi Vanvitelli, Naples, Italy

**Keywords:** diabetic retinopathy, neuroprotection, vitreous, curcumin, homotaurine, vitamin D3, pro-inflammatory cytokines

## Abstract

**Purpose:** To determine the levels of pro-inflammatory cytokines and soluble mediators (TNF-α, IL6, IL2, and PDGF-AB) in 28 vitreous biopsies taken from patients with proliferative diabetic retinopathy (PDR) and treated with increasing doses of curcumin (0. 5 and 1 μM), with or without homotaurine (100 μM) and vitamin D3 (50 nM).

**Materials and Methods:** ELISA tests were performed on the supernatants from 28 vitreous biopsies that were incubated with bioactive molecules at 37°C for 20 h. The concentration of the soluble mediators was calculated from a calibration curve and expressed in pg/mL. Shapiro-Wilk test was used to verify the normality of distribution of the residuals. Continuous variables among groups were compared using the General Linear Model (GLM). Homoscedasticity was verified using Levene and Brown-Forsythe tests. *Post-hoc* analysis was also performed with the Tukey test. A *p* ≤ 0.05 was considered statistically significant.

**Results:** The *post-hoc* analysis revealed statistically detectable changes in the concentrations of TNF-α, IL2, and PDGF-AB in response to the treatment with curcumin, homotaurine, and vitamin D3. Specifically, the *p*-values for between group comparisons are as follows: TNF-α: (untreated vs. curcumin 0.5 μM + homotaurine 100 μM + vitamin D3 50 nM) *p* = 0.008, (curcumin 0.5 μM vs. curcumin 0.5 μM + homotaurine 100 μM + vitamin D3 50 nM) *p* = 0.0004, (curcumin 0.5 μM vs. curcumin 1 μM + homotaurine 100 μM + vitamin D3 50 nM) *p* = 0.02, (curcumin 1 μM vs. curcumin 0.5 μM + homotaurine 100 μM + vitamin D3 50 nM) *p* = 0.025, and (homotaurine 100 μM + vitamin D3 50 nM vs. curcumin 0.5 μM + homotaurine 100 μM + vitamin D3 50 nM) *p* = 0.009; IL2: (untreated vs. curcumin 0.5 μM + homotaurine 100 μM + vitamin D3 50 nM) *p* = 0.0023, and (curcumin 0.5 μM vs. curcumin 0.5 μM+ homotaurine 100 μM + vitamin D3 50 nM) *p* = 0.0028; PDGF-AB: (untreated vs. curcumin 0.5 μM + homotaurine 100 μM + vitamin D3 50 nM) *p* = 0.04, (untreated vs. curcumin 1 μM + homotaurine 100 μM + vitamin D3 50 nM) *p* = 0.0006, (curcumin 0.5 μM vs. curcumin 1 μM + homotaurine 100 μM + vitamin D3 50 nM) *p* = 0.006, and (homotaurine 100 μM + vitamin D3 50 nM vs. curcumin 1 μM + homotaurine 100 μM + vitamin D3 50 nM) *p* = 0.022. IL6 levels were not significantly affected by any treatment.

**Conclusions:** Pro-inflammatory cytokines are associated with inflammation and angiogenesis, although there is a discrete variability in the doses of the mediators investigated among the different vitreous samples. Curcumin, homotaurine, and vitamin D3 individually have a slightly appreciable anti-inflammatory effect. However, when used in combination, these substances are able to modify the average levels of the soluble mediators of inflammation and retinal damage. Multi-target treatment may provide a therapeutic strategy for diabetic retinopathy in the future.

**Clinical Trial Registration :** The trial was registered at clinical trials.gov as NCT04378972 on 06 May 2020 (“retrospectively registered”) https://register.clinicaltrials.gov/prs/app/action/SelectProtocol?sid = S0009UI8&selectactio*n* = Edit&uid = U0003RKC&ts = 2&cx = dstm4o.

## Introduction

The number of people of all age groups who are visually impaired worldwide is estimated at 285 million; among these, 39 million are blind (1). Visual impairment is strongly associated with increasing age. In high-income regions of Central/Eastern Europe, diabetic retinopathy (DR) and glaucoma are the most important causes of vision loss (2). Glaucoma affects more than 70 million people worldwide (3) and it leads to progressive optic nerve degeneration, with a gradual loss of retinal ganglion cells (RGCs) (4, 5). The pathogenesis of glaucoma is not yet completely clarified (i.e., mechanical/ischemic insult, neuroinflammation, etc.) (6). Furthermore, although lowering intraocular pressure (IOP) has been clearly shown to decrease the progressive visual loss in most patients with glaucoma (7), there are some patients for whom IOP lowering is either insufficient, difficult to achieve, or associated with risks of adverse effects, especially when patients are treated with surgical procedures (8). Thus, other strategies are needed to reduce or reverse the progressive neurodegeneration, and this represents the rationale for therapies based on neuroprotection (9). By definition, neuroprotection is an effect that may result in rescue, recovery, or regeneration of the nervous system, its cells, structure, and function (10). In ophthalmology as well as for glaucoma, neuroprotection is also emerging as a therapeutic target for diabetic retinopathy (11).

Diabetic retinopathy (DR) is one of the most common complications of diabetes mellitus and is a leading cause of vision loss and blindness in the working-age population worldwide. Once considered solely as a microvascular disease, DR has been recognized as a neurodegenerative disease of the retina (12–13 effects of antioxidants). Progressive blindness is due to the long-term accumulation of pathological abnormalities in the retina of hyperglycemic patients. In the initial phase, non-proliferative diabetic retinopathy (NPDR) is almost asymptomatic, with the onset of microhemorrhagic and microischemic episodes and an increase in vascular permeability. Subsequently, the progression of the disease is accompanied by the onset of a chronic inflammatory state and neovascularization in a vicious circle that feeds and determines the accumulation of damage to the retina through hypoxia, oxidative stress, and widespread neurodegeneration. Among the metabolites, hyperglycemia is known to be the major factor activating several metabolic pathways that are harmful for the retina (12). Moreover, an increased level of glutamate has been reported in both the retina and vitreous of diabetic patients, suggesting a neurotoxic role of glutamate, which may damage retinal neurons, especially retinal ganglion cells, by excitotoxicity (12–15). Thus, glaucoma and diabetic retinopathy have in common the occurrence of a progressive neurodegeneration. In fact, several studies have shown that there is an overexpression of excitatory proteins, such as glutamate and NMDA, in the retina and vitreous in glaucoma, diabetic retinopathy, and multiple animal models of retinal ischemia (16, 17). In proliferative diabetic retinopathy (PDR), vitreous humor undergoes structural and molecular changes as well as changes in its composition, which play a pivotal role in supporting the disease progression (18). The vitreous is a transparent, gel-like structure of 4 mL in volume, which fills the space between the lens and the retina (19). It is composed of 98-99% of water, with traces of cations, ions, proteins (mainly collagen), and polysaccharides such as hyaluronic acid (20). In PDR patients undergoing pars plana vitrectomy, vitreous samples are characterized by altered levels of bioactive molecules, with pro-angiogenic, pro-inflammatory, and neuromodulatory activities (19). This clearly demonstrates that the vitreous acts as a reservoir of soluble signaling mediators that may exacerbate retinal damage. On the other hand, the vitreous obtained from patients with PDR can be a powerful tool to evaluate the anti-angiogenic/anti-inflammatory activity of new biomolecules that could be potential candidates for the treatment of diabetic vitreoretinopathy. Currently, PDR is treated with laser photocoagulation, vitreoretinal surgery, or intravitreal injection of drugs targeting the vascular endothelial growth factor (VEGF) and steroid agents (21). However, although these protocols are effective in the short term, they cause side effects and are indicated only for the advanced stages of the disease.

Thus, non-invasive, non-destructive, and longer-duration treatment options are also needed (22). Recently, research efforts have been made to identify neuroprotective compounds that are able to prevent visual field loss and preserve visual function. A promising alternative for the treatment of early-stage NPDR comes from nutraceuticals. In fact, *in vitro* and *in vivo* studies have revealed that a variety of nutraceuticals offers important antioxidant and anti-inflammatory effects that can counteract the first diabetes-driven molecular events that cause vitreoretinopathy, acting as upstream regulators of the disease (23). Based on the results of several investigations, it is reasonable to assert that a single constituent that affects one target has limited efficacy in preventing the progression of multifactorial diseases. A large body of research revealed that the use of a combination of compounds with synergistic multitarget effects may offer a more powerful approach for the prevention of diseases, including retinal neurodegeneration (24–27). In experimental models, it has been shown that the co-treatment of citicoline and homotaurine has a direct neuroprotective effect on primary retinal cells exposed to glutamate toxicity and high glucose (HG) levels (28). Glutamate-induced excitotoxicity is implicated in the pathophysiology of several degenerative diseases of the retina, including glaucoma. Moreover, HG-induced neurotoxicity is a characteristic of diabetic retinopathy (29, 30). Curcumin, a yellowish non-flavonoid polyphenol that constitutes the main active compound of *Curcuma longa*, is widely known for its antioxidant and anti-inflammatory properties (31–33). Many studies have also described its marked protective effect on retinal cells against oxidative stress and inflammation (31–35). Lastly, vitamin D3 levels appear to be lower in diabetes mellitus type 2 patients, and this could have therapeutic implications (36). This study aimed to analyze the soluble mediators of inflammation and angiogenesis in the vitreous of patients with diabetic retinopathy treated with homotaurine, curcumin, and vitamin D3.

## Materials and Methods

The study was conducted at the Department of Medicine and Health Sciences “V. Tiberio” of Molise University, Campobasso (Italy), in accordance with the ethical principles of the Declaration of Helsinki. The CTS (technical scientific committee) of the Department approved the study protocol (registered at clinicaltrials.gov, identifier NCT0437897). All the study participants provided written informed consent. This was a prospective study including 28 eyes of 28 patients consecutively enrolled from September 16, 2019 to December 16, 2019. The patients were scheduled to undergo a 23-gauge, three-port pars plana vitrectomy for retinal detachment, and all the patients completed the study. Inclusion criteria were age ≥18 years, patients with diabetic retinopathy requiring vitrectomy, and willingness to participate in the study following the indications provided. The exclusion criteria were previous vitrectomy in the study eye, previous buckle surgery, previous intravitreal injection, concurrent retinovascular or other ocular inflammatory disease, history of ocular trauma and concomitant intake of any topical or systemic NSAID or corticosteroid therapy, and presence of systemic inflammations. All phakic patients were operated with phacoemulsification of the crystalline lens plus intraocular lens (IOL) implant at the time of vitrectomy to allow a careful cleaning of the vitreous base. Vitrectomy surgery was performed using a 23-gauge transconjunctival system; no triamcinolone was used during any step of the surgery.

After the removal of the posterior hyaloids, the vitreous base was thoroughly removed. All the visible proliferative vitreoretinopathy (PVR) membranes were dissected, and relaxing retinotomies were performed. The retinal periphery was inspected for retinal breaks that were marked with endodiathermy, after which the retina was reattached using perfluorocarbon liquid and air. Three rows of endolaser treatment were applied behind the posterior vitreous base in all the patients (200 spots, 200–250 mW according to retinal pigmentation). All the patients in both groups were prescribed topical dexamethasone (six times per day) and homatropine (two times per day).

At the beginning of the surgery, 0.5–1.0 mL of undiluted aqueous was removed, and samples were immediately frozen and stored at −80°C until analysis. This procedure was used to prevent the vitrectomy intervention itself from generating or altering the expression of cytokines and endothelial growth factors or the BSS (balanced salt solution) from diluting the vitreous.

TREATED GROUP: Twenty-eight portions of vitreous samples from 28 eyes of patients undergoing vitrectomy for diabetic retinopathy complications, incubated with curcumin, homotaurine, and vitamin D3. The substances were treated with increasing doses of curcumin (Cureit®) (0.5 μM and 1 μM), with or without homotaurine (100 μM) and vitamin D3 (50 nM), to evaluate a possible synergistic effect on the expression of inflammatory cytokines.

CONTROL GROUP: The same fractions of vitreous samples (*n* = 28) were evaluated for the expression of oxidative biomarkers, inflammatory cytokines, and metalloproteinases, without any treatment.

PRIMARY ENDPOINT: Evaluation of the anti-inflammatory effect of curcumin, homotaurine, and vitamin D3 on the expression of inflammatory cytokines in human vitreous samples of patients with PDR.

### Reagents

Cholecalciferol (vitamin D3) and 3-Amino-1-propanesulfonic acid (homotaurine) were purchased from Sigma-Aldrich, whereas Cureit® curcumin was provided by Fisher Chemicals Aurea Biolab. Curcumin and vitamin D3 were first dissolved in DMSO (dimethyl sulfoxide) to final concentrations of 250 and 25 mM, respectively. Homotaurine was resuspended in PBS to a final concentration of 500 mM.

### ELISA Assays

Curcumin is a well-known bioactive molecule, largely employed in supplement formulation due to its anti-inflammatory properties. To evaluate its role in the regulation of the levels of pro-inflammatory soluble mediators in the vitreous fluid obtained from 28 patients with PDR, samples were exposed to curcumin at different concentrations for 24 h with or without homotaurine and vitamin D3. Subsequently, IL6, IL2, TNF-α, and PDGF-AB were measured by ELISA assays in 50 μl of diluted samples from different conditions.

Vitreous biopsies were thawed and centrifuged. Afterwards, 50 μL of vitreal fluid from each patient were aliquoted into 96-well plates and incubated for 24 h at 37°C in 100 μL HBSS (Hank's Balanced Salt Solution) with curcumin at different concentrations (0.5 and 1 μM) and with or without 100 μM homotaurine and 50 nM vitamin D3. Controls were exposed to HBSS containing DMSO. The day after, samples were diluted twice in sample diluent and cytokines were measured by ELISA assay.

Quantitative detection of soluble mediators in vitreal biopsies was performed using sandwich ELISA kits with High Sensitivity. IL2 and TNF-α were measured by Human Pre-coated ELISA Kit (BIOGEMS-PEPROTECH), whereas IL6 and PDGF-AB were detected using PicoKine ELISA Kits (Boster Biological Technology). All kit reagents, samples, and standards were prepared according to the manufacturer's instructions. The measured optical density was read at 450 nm and was directly proportional to the concentration of human recombinant proteins in the standards or samples. The concentration of soluble mediators was calculated from a calibration curve and expressed as pg/mL. Each experimental point was replicated three times, and absolute levels of IL6, IL2, TNF-α, and PDGF-AB were measured by ELISA. Subsequently, average levels of soluble mediators measured in treated vitreous were expressed as a percentage of the baseline level, considering the control aliquot from the same patient as baseline. According to manufacturer's instructions, optimal detection ranges were 15.6–1,000 pg/mL (sensitivity <0.1 pg/mL) for IL2; 15.6–1,000 pg/mL (sensitivity <1 pg/mL) for TNF-α; 4.69–300 pg/mL (sensitivity <0.3 pg/mL) for IL6; and 31.2–2,000 pg/mL (sensitivity <2 pg/mL) for PDGF-AB. Only the mean IL6 levels in vitreous biopsies were found to be close to the lower limit of detection, however, we could still measure significative optical densities in our experimental conditions.

### Gene Expression

To verify whether curcumin in combination with vitamin D3 and homotaurine in the vitreal fluid could exert an anti-inflammatory and anti-angiogenic effect, we monitored the expression of pro-inflammatory genes and mitogen-activated genes in an immortalized cell line exposed for 24 h to vitreal biopsies from patients with diabetic retinopathy together with curcumin, vitamin D3, and homotaurine or not. A subset of four vitreous was used in this experiment.

### Sample Size

The sample size was estimated with a suitable macro developed in the SAS language. We conducted a pilot study from which we derived the Mean Square Error (MSE). In order to make the data less variable, we applied the logarithmic transformation that made the residuals normal. The General Linear Model (GLM) provided the standard deviations (square root of MSE) necessary to perform the calculation of the Sample Size (±0.29, ±0.18, ±0.18, ±0.32, respectively, for PDGF-AB, IL2, IL6, and TNF-α).

Considering the following differences, on a logarithmic scale, *d* = 0.32, 0.13, 0.11, and 0.29, which correspond to a reduction of 52.1, 32.4, 22.4, and 48.7% relative to PDGF-AB, IL2, IL6, and TNF-α, we obtained with a power of 80% and α = 0.05 the following sample sizes: *n* = 19, 26, 61, and 28 subjects, taking into account the correction for multiple comparisons. For clinical purposes, we considered *n* = 28 as the final sample size.

### Statistical Analysis

Continuous variables (PDGF-AB, IL2, IL6, and TNF-α levels) were expressed as mean ± SD. Shapiro-Wilk test was used to verify the normality of distribution of the residuals.

To make the residuals normal, we applied suitable mathematical functions in order to respect the Gauss condition.

The continuous variables (PDGF-AB, IL2, IL6, and TNF–α levels) were compared among groups (untreated, curcumin 0.5 μM, curcumin 1 μM, homotaurine 100 μM + vitamin D3 50 nM, curcumin 0.5 μM + homotaurine 100 μM + vitamin D3 50 nM, and curcumin 1 μM + homotaurine 100 μM + vitamin D3 50 nM) using the GLM (General Liner Model) method. Homoscedasticity was verified by Levene and Brown-Forsythe tests. *Post-hoc* analysis was performed by the Tukey test.

A *p* ≤ 0.05 was considered statistically significant. The statistical analysis was performed using SAS v. 9.4 and JMP v. 15 (SAS Institute Inc., Cary, NC, USA).

## Results

Twenty-eight vitreous biopsies from 28 patients with PDR were analyzed. The mean age (± standard deviation) was 68.9 ± 7.8 years. Of the 28 included patients, 16 (57.1%) were males and 12 (42.9%) were females. Mean time (± standard deviation) since diagnosis of diabetes mellitus in these patients was 31.4 ± 8.7 years. The mean age (± standard deviation) at the time of vitrectomy was 68.9 ± 7.9 years old. In vitreous samples, the pro-inflammatory cytokines IL6, TNF-α, and IL2 and the angiogenic factor PDGF-AB were all detectable in the conditions of the sample. Mean IL6, IL2, TNF-α, and PDGF-AB levels in the vitreous of the patients are reported in Table 1.

**Table 1:** Levels of soluble mediators in vitreal biopsies from patients with diabetic retinopathy.

**Parameter**	**Untreated**	**Curcumin 0.5 μM**	**Curcumin 1 μM**	**Homotaurine 100 μM + Vitamin D3 50 nM**	**Curcumin 0.5 μM+ Homotaurine 100 μM + Vitamin D3 50 nM**	**Curcumin 1 μM + Homotaurine 100 μM + Vitamin D3 50 nM**	***p***
	**Mean ± SD (95% CI)**	**Mean ± SD(95% CI)**	**Mean ± SD (95% CI)**	**Mean ± SD(95% CI)**	**Mean ± SD (95% CI)**	**Mean ± SD(95% CI)**	
PDGF-AB^a^ (pg/mL)	842.68 ± 459.61 (664.50–1020.86)	780.43 ± 466.58(599.51–961.35)	657.58 ± 311.24 (536.89–778.26)	735.94 ± 466.40 (555.09–916.79)	538.32 ± 345.39 (404.40–672.25)	406.41 ± 213.85(323.48–489.33)	**0.0003**
IL2^b^ (pg/mL)	85.17 ± 47.33 (66.81–103.52)	81.95 ± 44.33(64.76–99.14)	63.00 ± 30.51 (51.17–74.83)	71.81 ± 41.05(55.89–87.73)	55.93 ± 26.73 (45.56–66.30)	60.21 ± 26.84(49.80–70.62)	**0.0005**
IL6^c^ (pg/mL)	16.71 ± 7.02 (13.99–19.43)	15.81 ± 6.11(13.44–18.18)	15.28 ± 5.88 (13.00–17.56)	15.96 ± 7.63(13.00–18.92)	13.78 ± 8.03 (10.67–16.90)	15.52 ± 8.39(12.27–18.78)	0.32
TNF-α^c^ (pg/mL)	112.56 ± 72.85 (84.32–140.81)	113.27 ± 50.20(93.81–132.74)	110.68 ± 82.95 (78.52–142.85)	108.61 ± 74.37 (79.78–137.45)	59.31 ± 42.09 (42.99–75.63)	66.52 ± 43.59(49.62–83.43)	**0.0001**

a *sqrt transformed*.

b *Inverse transformed*.

c *log_10_ transformed*.

*Post-hoc Analysis: PDGF-AB - (untreated vs. curcumin 0.5 μM + homotaurine 100 μM + vitamin D3 50 nM) p = 0.04; (untreated vs. curcumin 1 μM + homotaurine 100 μM + vitamin D3 50 nM) p = 0.0006; (curcumin 0.5 μM vs. curcumin 1 μM + homotaurine 100 μM + vitamin D3 50 nM) p = 0.006; (homotaurine 100 μM + vitamin D3 50 nM vs. curcumin 1 μM + homotaurine 100 μM + vitamin D3 50 nM) p = 0.022. IL2 - (untreated vs. curcumin 0.5 μM + homotaurine 100 μM + vitamin D3 50 nM) p = 0.0023; (curcumin 0.5 μM vs. curcumin 0.5 μM + homotaurine 100 μM + vitamin D3 50 nM) p = 0.0028. TNF-α - (untreated vs. curcumin 0.5 μM + homotaurine 100 μM + vitamin D3 50 nM) p = 0.008; (curcumin 0.5 μM vs. curcumin 0.5 μM + homotaurine 100 μM + vitamin D3 50 nM) p = 0.0004; (curcumin 0.5 μM vs. curcumin 1 μM + homotaurine 100 μM + vitamin D3 50 nM) p = 0.02; (curcumin 1 μM vs. curcumin 0.5 μM + homotaurine 100 μM + vitamin D3 50 nM) p = 0.025; (homotaurine 100 μM + vitamin D3 50 nM vs. curcumin 0.5 μM + homotaurine 100 μM + vitamin D3 50 nM) p = 0.009. Bold values are those statistically significant*.

**Figure 1:**
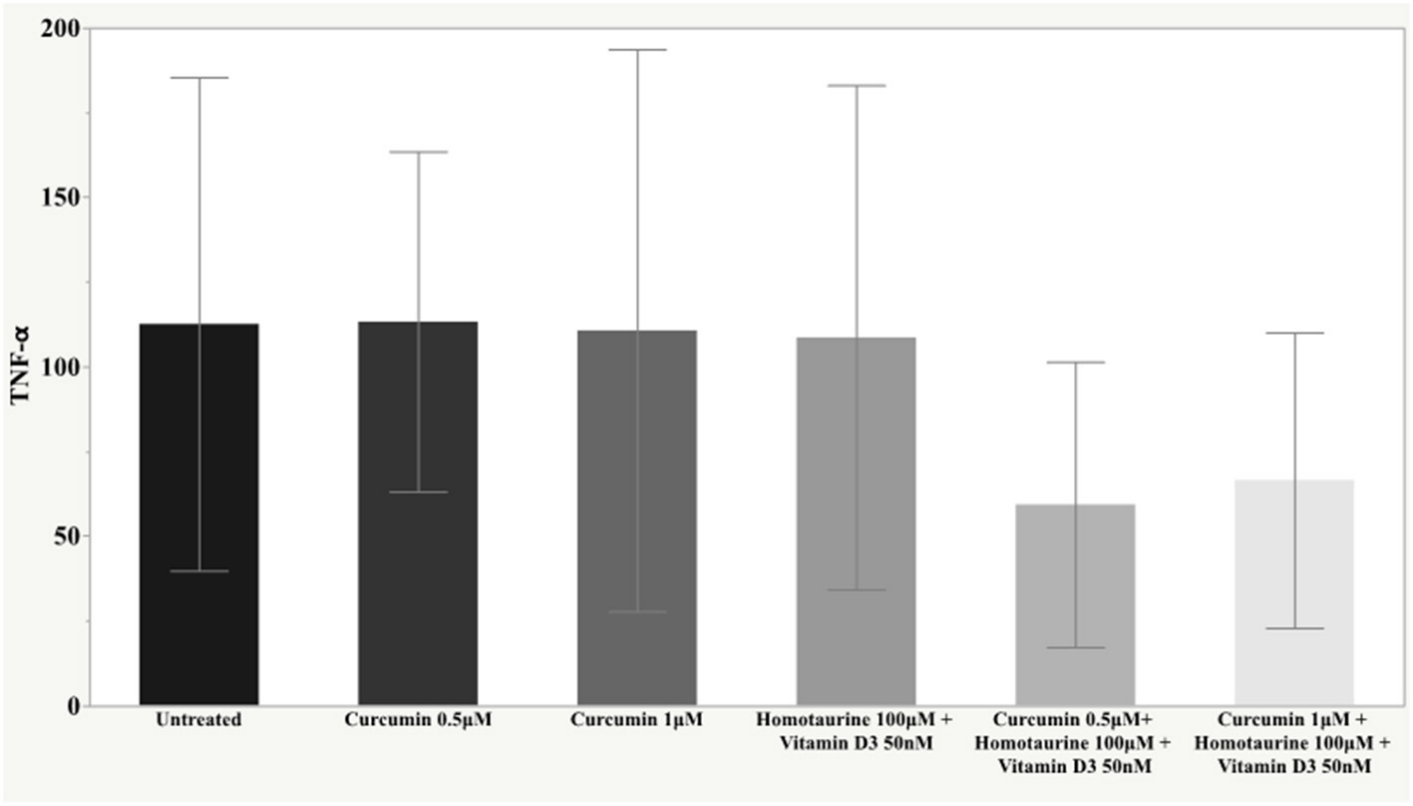
Histogram of the mean and standard deviations of TNF-α (pg/ml) by experimental groups.

**Figure 2:**
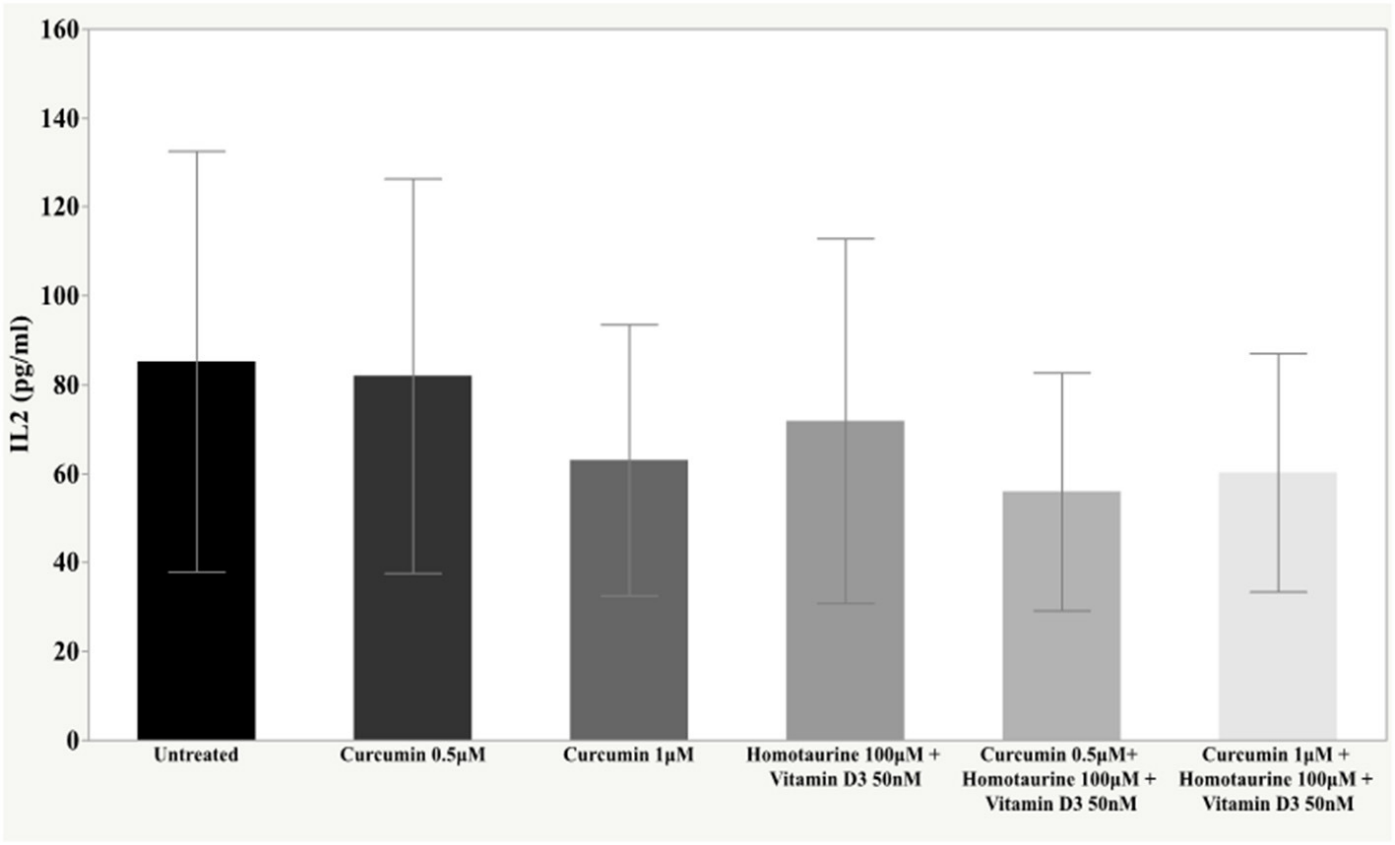
Histogram of the mean and standard deviations of IL2 by experimental groups.

**Figure 3:**
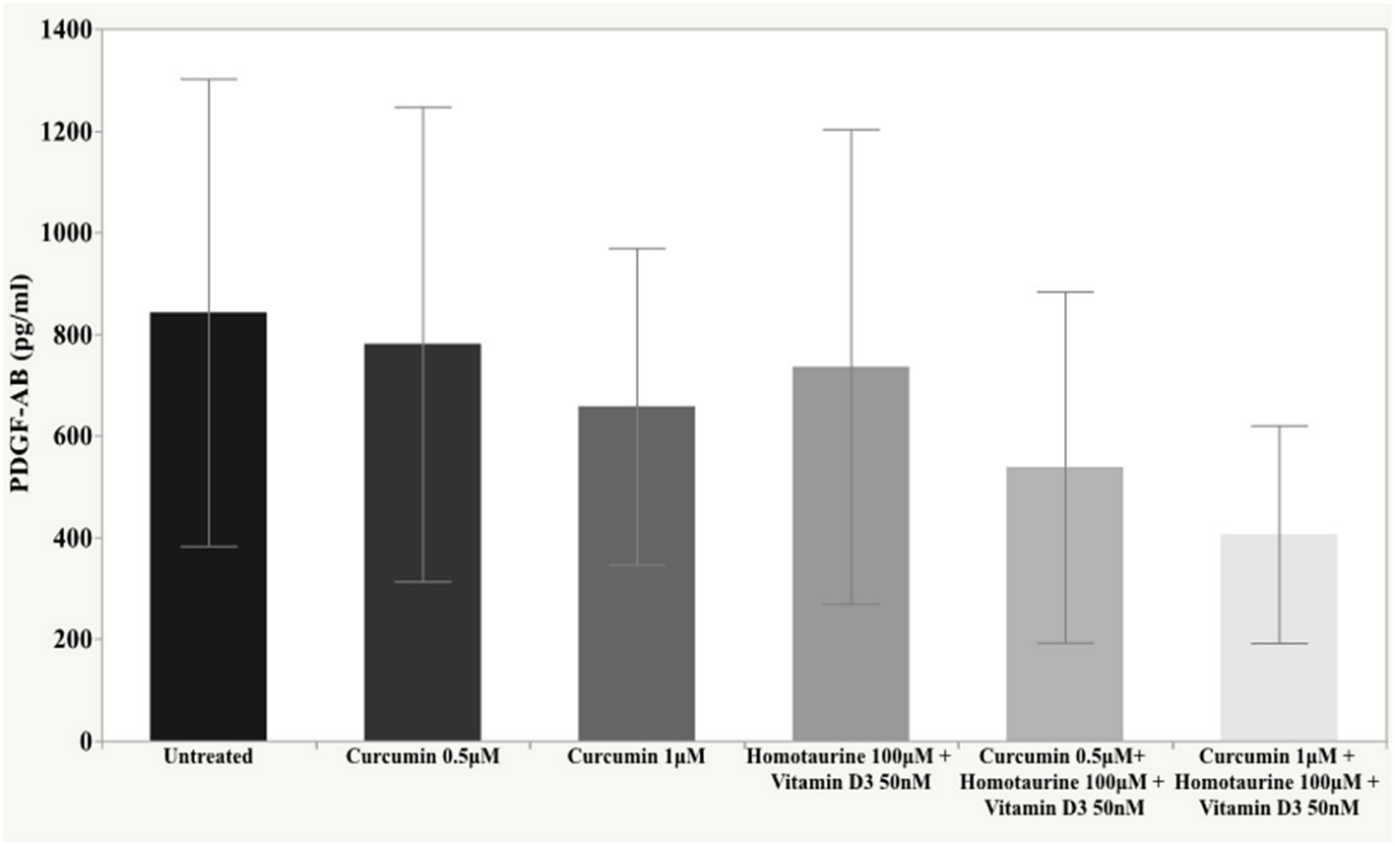
Histogram of the mean and standard deviations of PDGF-AB by experimental groups.

**Figure 4:**
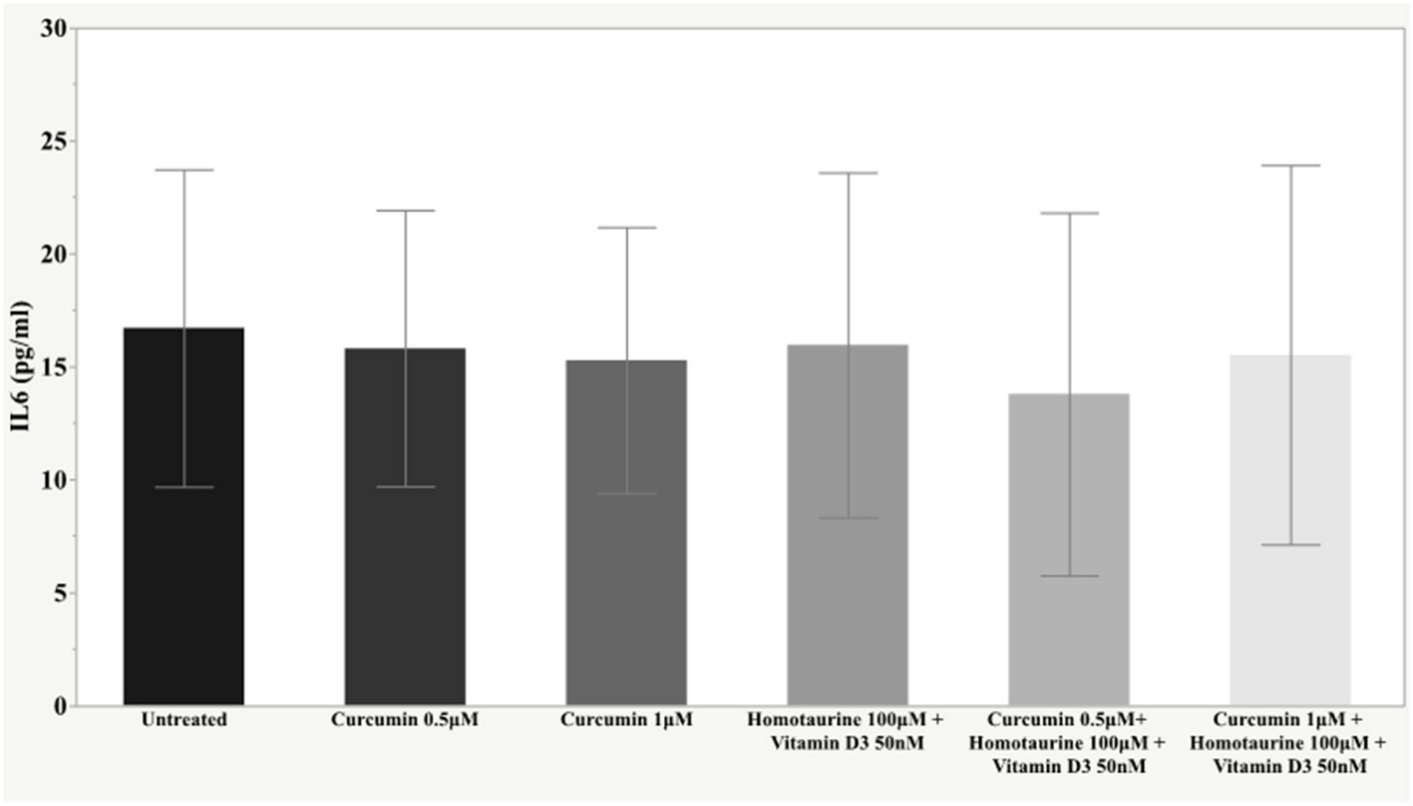
Histogram of the mean and standard deviations of IL6 by experimental groups.

The *post-hoc* analysis revealed statistically detectable changes in the concentration of TNF-α, IL2, and PDGF-AB in response to treatment with curcumin, homotaurine, and vitamin D3. Specifically, the p-values for between group comparisons are as follows: TNF-α: (untreated vs. curcumin 0.5 μM + homotaurine 100 μM + vitamin D3 50 nM) *p* = 0.008, (curcumin 0.5 μM vs. curcumin 0.5 μM + homotaurine 100 μM + vitamin D3 50 nM) *p* = 0.0004, (curcumin 0.5 μM vs. curcumin 1 μM + homotaurine 100 μM + vitamin D3 50 nM) *p* = 0.02, (curcumin 1 μM vs. curcumin 0.5 μM + homotaurine 100 μM + vitamin D3 50 nM) *p* = 0.025, and (homotaurine 100 μM + vitamin D3 50 nM vs. curcumin 0.5 μM + homotaurine 100 μM + vitamin D3 50 nM) *p* = 0.009 (Figure 1); IL2: (untreated vs. curcumin 0.5 μM + homotaurine 100 μM + vitamin D3 50 nM) *p* = 0.0023 and (curcumin 0.5 μM vs. curcumin 0.5 μM + homotaurine 100 μM + vitamin D3 50 nM) *p* = 0.0028 (Figure 2); PDGF-AB: (untreated vs. curcumin 0.5 μM + homotaurine 100 μM + vitamin D3 50 nM) *p* = 0.04, (untreated vs. curcumin 1 μM + homotaurine 100 μM + vitamin D3 50 nM) *p* = 0.0006, (curcumin 0.5 μM vs. curcumin 1 μM + homotaurine 100 μM + vitamin D3 50 nM) *p* = 0.006, and (homotaurine 100 μM + vitamin D3 50 nM vs. curcumin 1 μM + homotaurine 100 μM + vitamin D3 50 nM) *p* = 0.022 (Figure 3). IL6 levels were not affected by any treatment (Figure 4).

**Figure 5:**
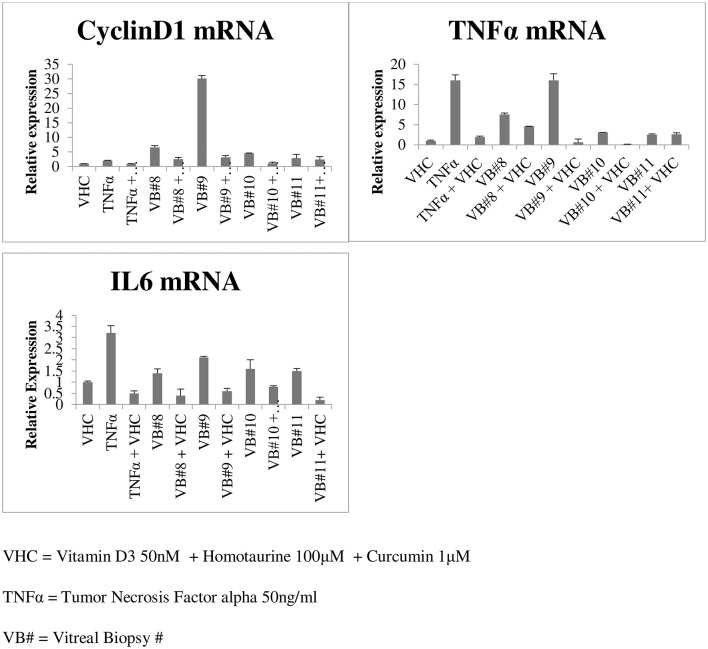
Gene expression in the vitreous of diabetic retinopathy patients.

Gene expression performed on four vitreous biopsies demonstrated that vitreal fluids can induce the cyclinD1 gene and the pro-inflammatory cytokine genes TNFα and IL6 on human HEK293 cells; contrarily when vitreal fluids were used in combination with curcumin, vitamin D3, and homotaurine such levels were down-regulated (Figure 5).

## Discussion

Intravitreal corticosteroids and anti-VEGF agents have become the first-line therapy for diabetic macular edema and PDR (37). However, in clinical practice, the use of anti-VEGF is not always applicable, due to the requirement of frequent injections and poor patient compliance. In fact, laser photocoagulation still plays an important role in the treatment of DR. However, in spite of a range of treatments such as those aforementioned, many patients with DR do not respond well to current approaches. Thus, there is still a need for more effective treatments, and biomarkers may help to gain knowledge of DR and contribute to the development of novel clinical strategies to prevent vision loss in people with diabetes (38). Optimization of current treatment therapies with regard to the number of intravitreal injections, dosage, and duration as well as of strategies for combination therapy is of great importance to improve the quality of life of patients with DR (37) and to enhance their chances of visual recovery.

The presence of a chronic systemic low-grade inflammation is characteristic of diabetes mellitus, contributing to the worsening of the inflammatory process that occurs in the eye (13, 22, 39–41). In DR, oxidative stress is promoted by several mechanisms, including the pathway of polyols (41–43), vascular dysfunction, production of pro-inflammatory cytokines (TNF-α, IL6, and interleukin-1 beta) and protein kinase C, accumulation of advanced glycation products, activation of the renin-angiotensin-aldosterone system, increment of growth factors, and leukostasis (44–53). Regarding cytokines, several studies have shown increased levels of pro-inflammatory cytokines in the vitreous and retina of diabetic patients and animals (53, 54). Indeed, intravitreal concentrations of major pro-inflammatory cytokines and chemokines, including IL1β, TNF-α, IL6, and IL8, are markedly upregulated in the vitreous of patients with PDR (55–57). Furthermore, an increase of TNF-α, IL8, and soluble IL2 receptor was observed in the progression from DR to PDR (58). It is clear that inflammation plays a key role in DR and, for this reason, the inhibition of the inflammatory pathway could be an interesting treatment option for DR (44, 45, 58, 59).

It is well known that the changes occurring in the retina are closely linked to biochemical changes in the vitreous humor (57, 60, 61). In fact, the vitreous has metabolic activity, and although it is considered as an acellular structure, phagocytic mononuclear hyalocytes and other cellular components are found in its different regions (62). Moreover, due to its proximity to the retina, the vitreous can undergo structural and biochemical changes that reflect the pathophysiological processes occurring in the retinal tissue (19, 61).

Our study highlighted the ability of curcumin to reduce cytokine levels in the vitreous of diabetic patients. We also observed an additional anti-inflammatory effect when curcumin was combined with homotaurine and vitamin D3, suggesting that these molecules can regulate the inflammatory network between the vitreous and retina at different levels. This effect is confirmed by the gene expression experiment which demonstrated that the combination of curcumin, vitamin D3, and homotaurine down-regulate the cyclinD1 gene and the pro-inflammatory cytokine genes TNFα and IL6 expression. It is well known that the synergism of curcumin with other bioactive molecules like those in turmeric makes a positive impact by generating high concentration of “free curcuminoids” in the blood plasma, as shown in previous studies (63). Moreover, homotaurine has been proven effective in reducing proinflammatory cytokines in synergy with other compounds. This result adds to the growing body of literature showing neuroprotective effects of homotaurine (30). Furthermore, the association of curcumin and homotaurine with vitamin D3 has also proved to be successful, confirming the results of numerous studies that have identified vitamin D as having a key role in diabetes. Vitamin D deficiency has been shown to impair insulin synthesis and secretion in animal models of diabetes (64, 65). Vitamin D3 decreases diabetes induced Reactive Oxygen Species (ROS) and exerts protective effects against retinal vascular damage and cell apoptosis in association with the inhibition of the ROS/TXNIP/NLRP3 inflammasome pathway (66, 67).

In our study, only IL6 levels showed no significant changes in response to treatment with the various compounds tested. This result could be due to the cross-talk between IL1β and IL6 signaling, more precisely due to the inhibitory action of IL1β on IL6 signaling, as has been reported by Shen et al. (68).

Finally, the identification of additional biomarkers in DR might lead to potential therapeutic targets and additive treatment options to improve metabolic control and neuroprotection in the context of individual customized therapy. This would maximize the patient's outcomes, with less collateral effects, leading to a reduction in the number of treatments, and enabling the control of lateral effects. In the DR treatment, anti-angiogenic therapy and anti-inflammatory agents could be used in combination, possibly simultaneously, to reduce the number of injections, risks, and costs (44).

## Conclusion

These findings confirm that pro-inflammatory cytokines and angiogenetic factors are associated with inflammation and angiogenesis, which synergistically contribute to the pathogenesis of DR. Our results underline that a multi-target treatment may provide a therapeutic strategy for DR treatment in the future. Natural anti-inflammatory compounds play an important role through their ability to reduce cytokine levels and regulate the inflammatory network (40) and reduce the rate of administration of anti-neovascularization agents, leading to an improvement in the quality of life of these patients.

## Data Availability Statement

The original contributions presented in the study are included in the article/supplementary materials, further inquiries can be directed to the corresponding author/s.

## Ethics Statement

The study was conducted at the Department of Medicine and Health Sciences V. Tiberio of Molise University, Campobasso (Italy), in accordance with the ethical principles of the Declaration of Helsinki. The CTS (technical scientific committee) of the Department approved the study protocol (registered at clinicaltrials.gov, identifier NCT0437897). All the study participants provided written informed consent.

## Author Contributions

MF and CC contributed to study concept and design, data analyses, interpretation of data, drafting of the manuscript, critical review of the manuscript, and study supervision. PV, TZ, LV, MR, and Rd'O performed acquisition of data, data analyses, interpretation of data, drafting of the manuscript, critical review of the manuscript. SB and GC conducted the study per protocol, interpretation of data, drafting of the manuscript, critical review of the manuscript. GC contributed to interpretation of data and statistical analysis. All authors contributed to the article and approved the submitted version.

## Conflict of Interest

The authors declare that the research was conducted in the absence of any commercial or financial relationships that could be construed as a potential conflict of interest.
